# Protection against Amyloid-β Oligomer Neurotoxicity by Small Molecules with Antioxidative Properties: Potential for the Prevention of Alzheimer’s Disease Dementia

**DOI:** 10.3390/antiox11010132

**Published:** 2022-01-07

**Authors:** Wataru Araki, Fuyuki Kametani

**Affiliations:** 1 Department of Neurology and Neurological Science, Tokyo Medical and Dental University, Bunkyo-ku, Tokyo 113-8510, Japan; 2 Department of Neurophysiology, National Institute of Neuroscience, National Center of Neurology and Psychiatry (NCNP), Kodaira, Tokyo 187-8502, Japan; 3 Department of Brain and Neuroscience, Tokyo Metropolitan Institute of Medical Science, Setagaya-ku, Tokyo 156-8506, Japan; kametani-fy@igakuken.or.jp

**Keywords:** amyloid beta, Alzheimer’s disease, neurotoxicity, oligomer, oxidative stress, small molecule

## Abstract

Soluble oligomeric assemblies of amyloid β-protein (Aβ), called Aβ oligomers (AβOs), have been recognized as primary pathogenetic factors in the molecular pathology of Alzheimer’s disease (AD). AβOs exert neurotoxicity and synaptotoxicity and play a critical role in the pathological progression of AD by aggravating oxidative and synaptic disturbances and tau abnormalities. As such, they are important therapeutic targets. From a therapeutic standpoint, it is not only important to clear AβOs or prevent their formation, it is also beneficial to reduce their neurotoxicity. In this regard, recent studies have reported that small molecules, most with antioxidative properties, show promise as therapeutic agents for reducing the neurotoxicity of AβOs. In this mini-review, we briefly review the significance of AβOs and oxidative stress in AD and summarize studies on small molecules with AβO-neurotoxicity-reducing effects. We also discuss mechanisms underlying the effects of these compounds against AβO neurotoxicity as well as their potential as drug candidates for the prevention and treatment of AD.

## 1. Introduction

Alzheimer’s disease (AD) is a neurodegenerative dementia whose prevalence is the highest among dementia disorders. AD is neuropathologically characterized by the extracellular deposition of amyloid-β protein (Aβ) as senile plaques, and intracellular accumulation of abnormally phosphorylated tau protein as neurofibrillary tangle [1]. Although it is established that the Aβ pathology precedes the tau pathology, the mechanisms by which the former is linked to the latter have not been clearly elucidated.

The time-course of AD progression has been rigorously studied in recent years. These studies have revealed that Aβ accumulation starts one to two decades before the onset of AD symptoms and reaches a substantial level at the mild cognitive impairment (MCI) stage before plateauing at the AD stage [2]. Tau deposition is already observable at the MCI stage and is correlated with synapse loss, which is also observed at this stage [2,3,4]. This and other accumulating evidence on the molecular pathology of AD contributes to the general acceptance of the idea that Aβ plays a primary role in the pathological sequence of AD [5]. 

Aβ is generated from its precursor, amyloid precursor protein (APP), through a two-step proteolytic process: (1) β-secretase (BACE1) or α-secretase (mainly ADAM10) cleaves APP; and (2) γ-secretase complexes, including presenilin 1 (PS1) or PS2, cleave the resultant BACE1- and α-secretase-generated C-terminal fragments to produce Aβ and p3 peptide, respectively [6]. Although the Aβ cascade hypothesis is widely accepted, more recent studies have proposed that the Aβ oligomer hypothesis accounts for the linkage between Aβ, tau, and synapse failure in AD [7,8]. According to this hypothesis, soluble assemblies of Aβ called Aβ oligomers (AβOs) exert neurotoxicity and synaptotoxicity, triggering the deleterious cascades that lead to the characteristic pathologies of AD. AβOs range in size from small to large, but it remains uncertain which species are the most toxic. This theory is supported by a large number of in vitro and in vivo studies including ours, which have collectively demonstrated that AβOs induce various pathological alterations, including oxidative stress, mitochondrial dysfunction, synaptic deficits, apoptosis, abnormal alterations of tau, and cognitive disturbances [7,8,9]. 

**Figure 1:**
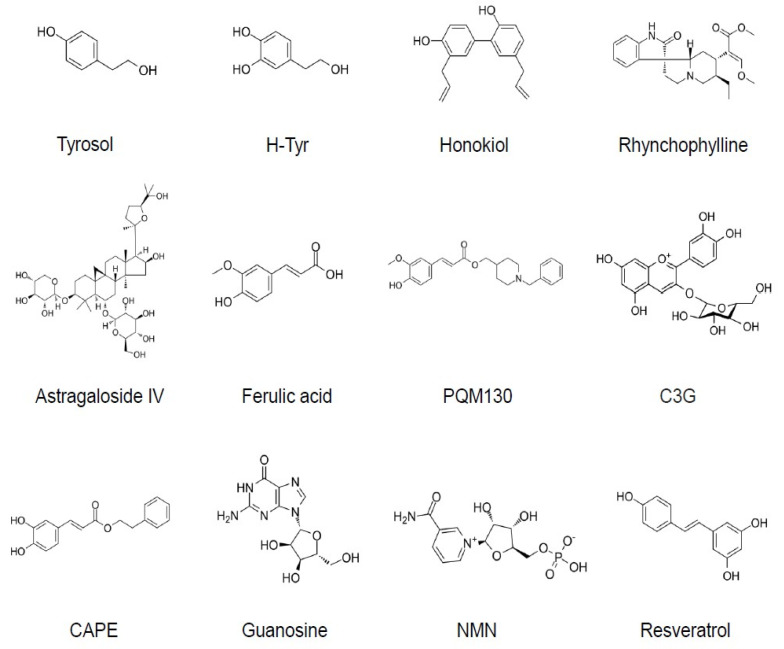
Chemical structures of the small molecules that can reduce AβO neurotoxicity. C3G (cyanidine 3-glucoside) is presented as a representative of anthocyanins. H-Tyr, hydroxytyrosol; CAPE, caffeic acid phenyl ester; NMN, nicotinamide mononucleotide.

In recent years, clinical trials of several drugs targeting Aβ, such as BACE1 inhibitors and anti-Aβ monocolonal antibodies, have failed, implicating the importance of targeting pathologies other than Aβ. Thus, more attention has been paid to drug candidates acting on other targets, including tau and neuroinflammation [10]. However, the Aβ oligomer hypothesis is still considered to be valid, and AβOs are attractive targets for the development of therapeutic candidates. In fact, strategies for removing or clearing AβOs using monoclonal antibodies specific for AβOs have already been developed. Inhibition of AβO formation is another plausible strategy [11,12,13]. In addition to these approaches, it should also be possible to directly target the neurotoxicity of AβOs. In fact, recent studies have revealed that the neurotoxicity of AβOs can be reduced by small molecules, most of which are from natural sources and possess anti-oxidative properties (Table 1, Figure 1).

In this mini-review, we briefly review the significance of AβOs and oxidative stress in AD and summarize studies on small molecules with protective activity against AβO neurotoxicity. We then discuss mechanisms through which these compounds exert their neuroprotective effects against AβOs and consider their potential as drug candidates for the prevention and treatment of AD dementia. Because our goal is to provide a concise review on this theme, we mainly focus on molecules whose effects have been analyzed in animal models.

## 2. AβOs and Oxidative Stress in AD

Under normal conditions, oxidative stress is managed by an array of physiological defense systems. However, in AD, such systems appear to be deranged, leading to the accumulation of oxidative damage. Aβ plays a major role in inducing oxidative stress, first through formation of radicals by virtue of Aβ interactions with metal ions such as cupper and iron, and second through oxidative stress generated as a result of the neurotoxic effects of AβOs [14]. Notably, oxidative stress is a pathological alteration characteristic of earlier AD stages, including MCI. Markers of oxidative stress include lipid peroxidation, protein carbonylation and nitration, and nucleic acid oxidation [14,15,16]. 4-hydroxynonenal (4-HNE), a major lipid peroxidation product and well-known oxidative stress marker, is also implicated in the pathological mechanism [17]. 

How AβOs trigger oxidative stress is currently unclear. Many in vitro and in vivo studies have shown that AβO exposure causes the production of reactive oxygen species (ROS), likely as a result of mitochondrial dysfunction. AβOs also induce Ca^2+^ dyshomeostasis, not only in the cytosol but also in mitochondria [18,19]. Specifically, AβOs have been shown to increase mitochondrial Ca^2+^ via the mitochondrial Ca^2+^ unipolar complex [19]. Associations between Aβ and mitochondrial proteins such as dynamin-related protein 1 (Drp1), cyclophilin D (CypD), and amyloid-binding alcohol dehydrogenase (ABAD) may also be involved in the mitochondrial dysfunction observed in AD [20]. Damaged mitochondria are selectively removed from cells through mitophagy (or mitochondrial autophagy), and the interaction between Aβ and mitochondria also affects the process of mitophagy [21]. Mitochondrial dysfunction and oxidative stress are mutually interactive processes. The consequences of oxidative stress include oxidative modifications of the enzymes involved in energy metabolism—modifications that could culminate in reduced glucose metabolism and decreased synthesis of ATP in the brain [15]. Furthermore, oxidative stress is linked to tau phosphorylation [20]. Thus, oxidative stress is closely associated with AβO neurotoxicity and plays a major role in the pathological mechanisms of AD. 

Among the most important defense mechanisms against oxidative stress is the Nrf2 (NFE2-like bZIP transcription factor 2) system. Under physiological conditions, Nrf2 is regulated by its cytoplasmic inhibitor, KEAP1. When activated, Nrf2 is translocated from the cytosol to the nucleus, where it binds to antioxidant response elements (ARE) to induce the expression of antioxidant and metabolic genes. In addition to KEAP1-dependent mechanisms, Nrf2 is also regulated by KEAP1-independent mechanisms, including negative regulation by glycogen synthase kinase-3β (GSK3β) [22]. Importantly, Nrf2 appears to be dysregulated in neurons in the AD brain, as levels of nuclear Nrf2 are decreased in the cortical and hippocampal tissues of AD cases [23], but it is unclear whether this dysregulation is related to Aβ. 

## 3. Small Molecules with Protective Activity against AβO Neurotoxicity

### 3.1. Tyrosol and Hydroxytyrosol 

Tyrosol (Tyr) and hydroxytyrosol (HT) are major antioxidative phenols in olive oils [24]. Tyr is also abundantly present in *Rhodiola rosea* [25]. Using a primary neuron culture system, Araki and others [26] have explored natural compounds for their potential to reduce the neurotoxicity of AβOs. They found that Tyr inhibits AβO-induced caspase-3 activation at a concentration of 5–10 μM, and also appeared to suppress the oxidative stress induced by AβOs. Thioflavin T (ThT) assays additionally showed that Tyr did not affect Aβ aggregation. These authors further examined the in vivo efficacy of Tyr in 5XFAD mice, showing that oral administration of Tyr (~12.5 mg/kg/d) for 12 or 20 weeks (from either 2 or 4–7 months of age) reversed the reduced expression of spinophillin, a dendritic synaptic protein, as well as the enhancement of 4-HNE in the hippocampus, suggesting that Tyr can alleviate synaptic and oxidative disturbances in this AD mouse model. Tyr treatment also modestly mitigated the spatial memory impairment of these mice, determined in the Barnes maze test. By contrast, Tyr administration did not affect Aβ accumulation in the brain.

Using the APP/PS1 mouse model of AD, Peng et al. [27] examined the effects of chronic oral administration of HT (5 mg/kg/d) from 3 months to 9 months of age. They showed that HT treatment attenuated oxidative stress responses in the brain and mildly improved cognitive function, as assessed by the Morris water maze (MWM) test without altering brain Aβ accumulation. Interestingly, they showed that HT normalized p-JNK/p-p38 signaling in the cerebral cortex of the mice and further demonstrated that HT can cross the blood–brain barrier (BBB). 

In another study using a mouse model in which AβOs and ibotenic acid (AβOi) were injected into the lateral ventricle [28], HT, administered orally for 14 days, attenuated spatio–cognitive deficits, as measured using the radial maze test. Notably, AβOi-induced abnormalities in PI3K/Akt1, ERK/MAPK/RSK2, JAK2/STAT3, and JNK/p38 signaling pathways were reversed by HT. A recent study also reported that oral administration of HT acetate (50 mg/kg) into APP/PS1 mice for 3 months (from 3.5 to 6.5 months old) attenuated apoptosis in the cortex and hippocampus and improved cognitive function, measured using T-maze and MWM tests [29]. 

A study using neuroblastoma N2a cells showed that Tyr and HT alleviated Aβ_25–35_-induced neurotoxicity via a mechanism that appeared to involve NF-κB signaling [30]. In an in vitro model of Parkinson’s disease (PD), Tyr treatment protected catecholaminergic cells from 1-methyl-4-phenylpyridinium (MPP^+^)-induced cell death, possibly through activation of the PI3K/Akt signaling pathway [31]. Tyr also delayed neurodegeneration in a *Caenorhabditis elegans* model of PD, possibly through antioxidative mechanisms [32]. Tyr and HT were shown to activate the Nrf2 pathway in some models [33,34], but it remains to be investigated whether activation of Nrf2 signaling is involved in their protective effects against AβOs. Notably, Tyr and tyrosol sulfate, a major metabolite of Tyr, have similar antioxidative and anti-inflammatory effects [35], implying that the action of Tyr is long-lasting.

### 3.2. Honokiol

Honokiol, a natural polyphenol isolated from *Magnolia officinalis*, has various neuromodulating effects, including neuroprotective effects against Aβ-induced neurotoxicity in PC12 pheochromocytoma cells [36,37]. In this model, honokiol prevented abnormal GSK3β and β-catenin signaling induced by Aβ [37]. Honokiol was further shown to have ameliorative effects in AβO-injected in vivo models [38]. Using an AβO intrahippocampal injection model, Wang et al. showed that honokiol, intraperitoneally administered for 14 days, improved spatial learning impairments in a dose-dependent manner in association with inhibition of neuronal loss and apoptosis in the CA1 region of the hippocampus. These researchers further presented evidence that honokiol (10–100 μM) prevented AβO-induced apoptosis in primary hippocampal neurons. Interestingly, they showed that honokiol inhibited ROS generation and the NF-κB signaling pathway in AβO-treated neurons.

Another study examined the effect of honokiol in PS1V97L transgenic mice and primary cultured neurons [39]. In the mouse model, intraperitoneal injection of honokiol for 3 months (at 6 to 9 months of age) improved cognitive deficits, measured using the MWM test. In cultured neurons, honokiol had protective effects on neuronal cell viability and ROS generation. This study further revealed that SIRT3 (sirtuin 3) levels were decreased in the mitochondrial fractions of AβO-treated neurons and those of the hippocampal tissues of PS1 transgenic mice, effects that were partially reversed by honokiol treatment. These results suggest that honokiol can attenuate mitochondrial dysfunction by regulating the activity of mitochondrial SIRT3.

Honokiol also downregulated BACE1, lowered Aβ deposition, suppressed neuroinflammation, and improved cognitive impairment in APP/PS1 transgenic mice. Interestingly, honokiol enhanced PPARγ function and its ameliorative effects were blocked by GW9662, a PPARγ antagonist [40].

### 3.3. Rhynchophylline

Rhynchophylline (Rhy) is a biologically active component present in *Uncaria rhynchophylla*, which possesses cardioprotective and neuroprotective effects [41]. Rhy has been shown to protect against AβO-induced cognitive impairment through two different pathways—antagonism of GluN2B-containing NMDA receptors (GluN2B-NMDARs) and activation of Nrf2/ARE pathways.

In a study investigating the first of these effects [42], AβOs were bilaterally injected into the hippocampus, followed by bilateral hippocampal injection of Rhy. Excitatory postsynaptic potentials (fEPSPs) were then recorded in the dentate gyrus region in vivo. These studies showed that AβO administration impaired long-term potentiation (LTP), an effect that was reversed by Rhy treatment. This impairment in LTP depended on activation of GluN2B-NMDARs, as evidenced by the fact that cotreatment with the GluN2B-selective antagonist, ifenprodil (Ifen), reversed the impairment. Furthermore, Rhy prevented AβO-induced cognitive impairment (MWM test), a protective effect that was recapitulated by Ifen. Collectively, these observations suggest that the protective effect of Rhy involves blocking GluN2B-NMDARs.

The study investigating the second of these two pathways used a similar mouse model [43]. In this case, Aβ_42_ was injected into the lateral ventricle, and after 3 days, Rhy (10 or 20 mg/kg) was administered intraperitoneally for 7 days. These authors showed that Aβ_42_ induced cognitive deficits, measured in the MWM test and Y-maze avoidance test, which were reversed by Rhy treatment. Furthermore, Aβ_42_ treatment induced oxidative stress in the hippocampus and frontal cortex, as indicated by elevated levels of ROS and malondialdehyde (MDA), and reduced levels of glutathione (GSH), all of which were reversed by Rhy treatment. They also showed that TUNEL-positive (apoptotic) cells increased in brain regions of Aβ-treated mice, and that Rhy treatment reduced the number of these cells, suggesting that Rhy can inhibit Aβ-induced apoptosis. Notably, Rhy treatment increased Nrf2 levels in total and nuclear extracts of brain tissues, and also significantly increased protein levels of the Nrf2 downstream targets, HO-1 (heme oxigenase-1), NAD(P)H quinone dehydrogenase 1 (NOQ1), and GCLM (glutathione cysteine ligase modulatory subunit). These results suggest that Rhy acts through Nrf2 activation to exert protective effects against Aβ-induced neurotoxicity.

### 3.4. Astragaloside IV

Astragaloside IV (AS-IV), a small-molecular-weight saponin isolated from *Radix astragali*, has been shown to act as a PPARγ (peroxisome proliferator-activated receptor gamma) agonist [44]. AS-IV also appears to have anti-oxidative and neuroprotective effects through Nrf2 activation [45]. A recent study by Wang et al. [46] showed that AS-IV has beneficial effects on AβO-induced neurotoxicity in vitro and in vivo. They demonstrated that AS-IV prevents AβO-induced death of neuronal HT22 cells, and that AS-IV suppresses the AβO-induced decrease in BDNF (brain-derived neurotrophic factor) by promoting PPARγ expression. They further examined the effect of AS-IV on memory impairment in Aβ-injected mice. In these experiments, AS-IV was administered by gavage for 1 week, followed by intrahippocampal injection of AβOs, after which mice received AS-IV or AS-IV plus the PPARγ antagonist GW9662 for 4 weeks. AS-IV suppressed AβO-induced neuronal loss and apoptosis in the hippocampus and ameliorated spatial memory impairment, measured using the MWM test. In agreement with the in vitro data, AS-IV treatment in vivo upregulated PPARγ expression and increased levels of BDNF mRNA and protein expression in mice. Thus, the PPARγ/BDNF signaling pathway is probably involved in the protective effect of AS-IV against the neurotoxicity of AβOs.

Notably, AS-IV also exerts inhibitory effects on BACE1 protein levels by suppressing BACE1 mRNA expression via PPARγ activation, an effect that leads to a reduction in Aβ levels and Aβ plaques in vitro and in vivo (in APP/PS1 mice) [44]. Thus, AS-IV appears to have multiple effects that beneficially modulate AD pathology.

### 3.5. Ferulic Acid and Related Compounds

Ferulic acid (FA), an antioxidant present in plant cell walls, has been suggested to have inhibitory effects on Aβ aggregation [47]. FA was shown to protect against Aβ_42_-induced oxidative stress and neurotoxicity in rat primary cortical neurons [48]. Although this study did not employ AβOs, another study showed that FA inhibited AβO-induced cell death and apoptosis in LAN5 neuroblastoma cells [49]. FA was also shown to decrease AβO-induced intracellular ROS generation and normalize depolarization of mitochondrial membrane potential; notably, these effects of FA were enhanced using FA entrapped in solid lipid nanoparticles. Modulation of ERK1/2 signaling appeared to be involved in the above effects. Because FA was shown to exert antioxidant effects through ERK1/2-mediated activation of Nrf2 in a PD model [50], the Nrf2 pathway may be involved in the protective effect of FA against AβOs. In addition, chronic oral FA administration was shown to decrease cerebral Aβ deposition and mitigate cognitive impairment in APP/PS1 transgenic mice [51,52].

PQM130, an FA-related molecule, is a unique compound synthesized by combining two subunits—the N-benzylpiperidine group present in donepezil and the feruloyl group present in FA [53]. PQM130 was shown to exert neuroprotective and antioxidative activities in human SH-SY5Y cells [54]. Moreover, in an in vivo model employing intracerebroventricular injection of AβOs in mice, PQM130 (0.5–1 mg/kg), administered intraperitoneally for 10 days after AβO injection, attenuated the oxidative damage, neuronal cell death (apoptosis), and neuroinflammation induced in the hippocampus by AβOs, possibly through modulation of pGSK3β and pERK1/2; it also ameliorated AβO-induced cognitive impairment [53].

### 3.6. Anthocyanins

Anthocyanins (Anthos) are polyphenolic flavonoids found in various plants that exhibit potent antioxidant activity [55]. Studies have shown that Anthos exert neuroprotective effects in various models. In particular, Korean black bean Anthos, in which cyanidin 3-glucoside (C3G) is the major component, were found to exert neuroprotective effects against Aβ_42_ in both in vitro and in vivo models [56]. A recent study examined whether the black bean Anthos are protective against AβO (Aβ_42_) neurotoxicity in APP/PS1 transgenic mice and HT22 cells [57]. In these in vivo experiments, Anthos (12 mg/kg/d) were administered intraperitoneally to the transgenic mice for 30 days (at 10–11 months of age). Antho treatment mitigated ROS production and oxidative stress and prevented neurodegeneration (apoptosis) in APP/PS1 mice as well as in AβO-exposed HT22 cells. Anthos also improved memory function in APP/PS1 mice in behavioral tests (MWM and Y-maze). Importantly, these studies also showed that treatment with Anthos upregulated p-PI3K/Akt/GSK3β(Ser9) signaling, nuclear translocation of Nrf2, and expression of its target proteins, HO-1 and GCLM, in both in vitro and in vivo models. Activated GSK3β induces downregulation of Nrf2, and GSK3β activation is prevented by phosphorylation at its serine-9 residue via activation of the PI3K/Akt signaling pathway. Thus, Anthos appear to provide antioxidant neuroprotection against the neurotoxicity of AβOs by acting through regulation of PI3K/Akt/GSK3β signaling to activate the Nrf2/HO-1 pathway,

A study by Song et al. [58] showed that oral administration of C3G for two months alleviated cognitive deficits in APP/PS1 mice, and C3G upregulated PPARγ in the nucleus in SH-SY5Y cells. C3G was also shown to attenuate the toxicity of Aβ_40_ via the Nrf2 signaling pathway in SH-SY5Y cells [59].

### 3.7. Caffeic Acid Phenyl Ester

Caffeic acid phenyl ester (CAPE), an antioxidative compound abundant in honeybee propolis, was shown to have beneficial effects against neuronal injuries [60]. In a recent study examining the effects of CAPE on AD pathology and cognitive functions in AβO-injected mice [61], CAPE, intraperitoneally administered for 10 days, significantly reversed spatial memory impairment, measured using the MWM test. CAPE also prevented AβO-induced caspase 9 activation and oxidative stress responses in the hippocampus. Notably, the anti-oxidative actions of CAPE in this AβO-injection model appeared to be mediated by activation of Nrf2 signaling and induction of HO-1. In addition, inflammatory activation of microglia and astrocytes was shown to be reduced by CAPE treatment. CAPE was also reported to inhibit LPS (lipopolysaccharide)-induced microglial activation in vitro and in vivo [62].

### 3.8. Guanosine

Guanosine (GUO), an endogenous guanine-based purine, exhibits neurotrophic and neuroprotective effects in various models [63]. It appears to exert these effects by reducing inflammation, oxidative stress, and glutamate excitotoxicity. An in vitro study using neuroblastoma SH-SY5Y cells showed that treatment of AβO-exposed cells with GUO (75 μM) prevented neuronal cell death (apoptosis) and ROS formation [64]. Another recent study investigated whether GUO protects against AβO neurotoxicity in an AβO-injection mouse model, showing that GUO (7.5 mg/kg) given orally 1 h before and 1, 3, and 6 h after intracerebroventricular injection of AβOs crosses the BBB and rescues short-term memory impairments, measured using the object-recognition test. GUO also rescued Ca^2+^ dyshomeostasis in hippocampal synaptosomes caused by AβOs [65]. The mechanisms underlying these protective effects of GUO remain to be clarified.

### 3.9. Nicotinamide Mononucleotide (NMN) and Nicotinamide

NMN is a precursor of nicotinamide adenine dinucleotide (NAD^+^), which is essential for energy metabolism and cellular functions. NAD^+^ has neuroprotective effects against several stimuli, including oxidative stress [66]. A study by Wang et al. [67] showed that treatment with NMN prevented neuronal cell death and inhibition of LTP in organotypic hippocampal slice cultures exposed to AβO. NMN also restored levels of NAD^+^ and ATP, and decreased ROS accumulation in AβO-treated hippocampal slices. They further found that intracerebroventricular injection of AβO-induced cognitive impairment in rats (assessed by the MWM test), which was significantly ameliorated by the intraperitoneal injection of NMN. Another study by Yao et al. [68] examined the therapeutic effects of NMN in APP/PS1 mice. NMN was subcutaneously applied every other day for 28 days. They showed that NMN treatment significantly decreased Aβ accumulation and inflammatory responses, and rescued cognitive impairments in the AD model mice. The inhibitory effect of NMN on Aβ pathology appeared to be attributable to the suppression of APP phosphorylation at Thr668 and the enhancement of non-amyloidogenic APP processing [68].

Nicotinamide is also a precursor of NAD^+^ and has been shown to protect against Aβ_42_- (not AβO) injection-induced oxidative stress, neuronal cell death, neuroinflammation, and memory dysfunction in mice [69]. In addition, administration of nicotinamide in the drinking water for 8 months reduced Aβ accumulation and ameliorated cognitive deficits in 3XTgAD mice (an animal model of AD) [70]. Preclinical trials of NAD^+^ precursors for AD models were systematically reviewed in a recent article by Wang et al. [66].

### 3.10. Other Compounds

#### 3.10.1. Resveratrol

SIRT1 plays a role in resistance to oxidative stress through interactions with molecules such as forkhead box O (FoxO) transcription factors and NF-κB [71]. Resveratrol (RSV), a SIRT1 activator, was shown to inhibit AβO cytotoxicity in SH-SY5Y cells [72] and to attenuate neuron viability loss and oxidative stress in cultured neurons exposed to AβOs [73]. RSV administered orally to APP/PS1 mice for 2 months (from the age of 4 months) reduced both senile plaques and oxidative stress responses compared with untreated mice [73]. The reduction in senile plaques may have resulted from the promotion of non-amyloidogenic APP processing through activation of ADAM10 by SIRT1 [74].

#### 3.10.2. Myricetin

Myricetin, a polyphenolic flavonoid present in plants such as grapes and berries, has many biological actions, including antioxidant, anti-inflammatory, and anti-bacterial effects [75]. Myricetin was also shown to inhibit Aβ oligomerization [76]. A recent study reported that myricetin prevented high molecular weight (HMW)-AβO-induced cell death and membrane disruption in SH-SY5Y cells [77]. Myricetin also suppressed HMW-AβO-induced mitochondria dysfunction, as demonstrated by reductions in membrane permeability transition and Mn-superoxide dismutase (SOD) and ATP generation, and increases in mitochondrial membrane potential and ROS generation. These findings suggest that myricetin prevents AβO-induced neurotoxicity through multiple antioxidant effects.

#### 3.10.3. 17 Oxo Sparteine and Lupanine

17 oxo sparteine and lupanine are quinolizidinic alkaloids that can be obtained from Cytisus scoparius. These alkaloids were shown to prevent AβO-induced toxicity in PC12 cells at a concentration of 0.03 μM [78]. They also prevented AβO-induced toxicity and increased the frequency of spontaneous Ca2+ transients, suggesting that these alkaloids enhance neural network synaptic activity. Furthermore, the neuroprotective effects elicited by both alkaloids were completely blocked by α-bungarotoxin, suggesting that their neuroprotective action is mediated by the nicotinic acetylcholine receptor.

#### 3.10.4. Esculetin

Esculetin (ESC) belongs to the coumarin family, which are phenolic compounds found in medicinal plants. ESC has been shown to have antioxidative and other beneficial activities in several disease models [79]. One study showed that ESC (20 μM) prevented AβO-induced death of neuronal SH-SY5Y cells and also counteracted AβO-induced formation of ROS [80]. Akt and ERK signaling pathways were suggested to be involved in the neuroprotection mediated by ESC. Interestingly, ESC appears to be a multifunctional compound, exhibiting inhibitory activity against both acetylcholine esterase and BACE1 [81].

## 4. Antioxidative and Other Mechanisms Involved in Small-Molecule-Mediated Prevention of AβO Neurotoxicity

**Figure 2:**
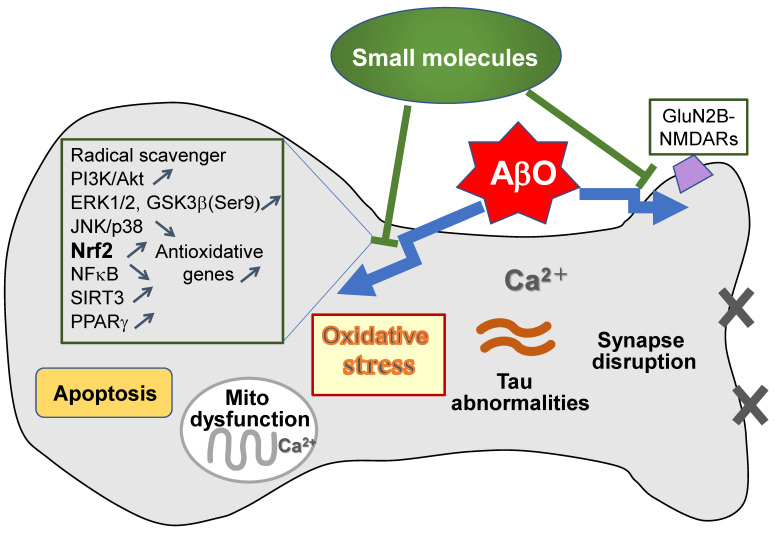
Mechanisms of action of small molecules with AβO-neurotoxicity-reducing effects. AβOs induce various pathological abnormalities, including oxidative stress, Ca^2+^ dyshomeostasis, mitochondrial (mito) dysfunction, apoptosis, synaptic disruption, and tau abnormalities, leading to cognitive impairment. These responses are ameliorated by application of the antioxidative small molecules described in the text. These molecules exert ameliorative effects through various mechanisms, including activation of PI3K/Akt and Nrf2 pathways, inhibition of the JNK/p38 pathway, and antagonism of GluN2B-NMDARs. Notably, most of the small molecules have Nrf2-stimulating activity.

Most of the above-mentioned small molecules that protect against AβO neurotoxicity have anti-oxidative properties (Table 1), suggesting the crucial involvement of antioxidative activity in the protection mechanisms. These antioxidative actions are mediated through two main mechanisms, depending on the characteristics of the molecules—direct elimination of ROS by acting as a free radical scavenger or a metal chelator [82], and enhanced expression of antioxidative genes regulated by activation of Nrf2 signaling. In fact, most of the small molecules described above have Nrf2-stimulating activity, and PI3K/Akt, GSK3β, and ERK1/2 pathways seem to be important for the activation of Nrf2 by these small molecules (Figure 2). Notably, the Nrf2 system appears to be dysregulated in AD brains [23]. Consistent with this, nuclear Nrf2 expression was shown to be reduced in the brains of APP/PS1 mice compared with wild-type mice [38,83], and overexpression of Nrf2 was shown to protect neurons against Aβ_42_ toxicity [84]. Thus, Nrf2 activation has been proposed as an attractive therapeutic strategy for AD [22].

In addition to their antioxidative actions, some of the molecules that reduce AβO-induced neurotoxicity likely promote cell survival and prevent cell death through PI3K/Akt and JNK/p38 pathways, respectively. This is consistent with the idea that abnormal JNK signaling is linked to Aβ-induced neuronal death [85].

Other signaling mechanisms involved in the protection against AβOs by small molecules include NF-κB, SIRT3, and PPARγ pathways (Figure 2). Activation of NF-κB, which is involved in inflammatory responses as a main regulator of the generation of inflammatory cytokines, is associated with neurodegeneration in the AD brain [86]. NF-κB, a heterodimer consisting of the subunits p50 and p65, is translocated to the nucleus upon release from its cytosolic negative regulator, IκB. Activation of MAPKs (ERK1/2, JNK, and p38) is intimately related to NF-κB signaling [86]. NF-κB p65 levels were shown to be elevated in Aβ-treated neuronal and glial cells and in neurons in the hippocampus and cortices of AD brains [87]. Based on these and other findings, inhibitors of NF-κB have been suggested to be useful for AD treatment [86]. In this context, some of the above-mentioned molecules, including Tyr, HT, and honokiol, have been shown to inhibit NF-κB signaling. In addition, it should be noted that Nrf2 and NF-κB pathways are interrelated [88].

SIRT3, a member of the Sir2 family of NAD^+^-dependent deacetylases, is a predominantly mitochondrial protein that plays important roles in regulating mitochondrial function and preventing oxidative stress through deacetylation of its substrates, including SOD2 (superoxide dismutase 2) and GDH (glutamate dehydrogenase) [89]. Among the small molecules described above, honokiol appears to rescue AβO-induced mitochondrial dysfunction by increasing mitochondrial SIRT3 levels.

The transcription factor PPARγ, which is known to regulate peripheral lipid and glucose metabolism, also plays roles in both glial cells and neurons [90]. PPARγ has a wide spectrum of functions, including regulation of mitochondrial function and antioxidant defense [90]. Interestingly, PPARγ appears to be associated with Nrf2 activation. PPARγ activation also upregulates Bcl-2, an anti-apoptotic member of the B-cell lymphoma-1 family in neurons, and protects neurons from Aβ-induced damage [91,92]. PPARγ upregulates BDNF [46] and suppresses BACE1 [93]. Notably, AS-IV appears to act as both a PPARγ agonist and Nrf2 activator.

In addition, blocking GluN2B-NMDARs appears to contribute to the protective action of Ryn against AβOs. This is consistent with the idea that the GluN2B-NMDAR is an important candidate receptor that mediates AβO-related neuronal dysfunction [94].

Recent evidence suggests that AβOs induce activation of microglia. Activated microglia release proinflammatory cytokines and neurotoxins, which could further trigger neuronal dysfunction and increase Aβ production [95]. Reactive astrocytes also contribute to neuroinflammation. Thus, inhibition of Aβ-induced neuroinflammation is important in the prevention of the pathological progression of AD. Some of the small molecules which exhibit anti-inflammatory properties are advantageous in this context. Although it is not clearly understood how they exert anti-inflammatory effects, the involvement of NF-κB signaling is suggested, as described above.

## 5. Issues in Translational Research and Future Perspectives

There are several issues regarding small molecules that prevent AβO neurotoxicity that should be taken into consideration in the context of translational research. The first is penetration across the BBB. Only a small number of such molecules have been shown to cross the BBB. As indicated by studies on FA, depending on the molecular properties of the specific compound, it may be important to increase BBB penetrance using lipid nanoparticles. The second issue is route of administration. Small molecules that can be administered orally are clearly advantageous with respect to clinical applications. From the standpoint of both BBB penetrance and administration route, Tyr and HT are ideal candidates for prophylaxis and/or treatment. The third issue relates to experimental models. The primary benefit of AβO-injection rodent models is assessment of short-term effects, whereas transgenic mouse models seem to be more appropriate for preclinical assessment of chronic beneficial effects. The fourth issue is how to determine appropriate doses for clinical application in humans. Resolving this issue is necessary for the design of clinical trial protocols and will require collecting more data on the pharmacokinetics and pharmacodynamics of specific compounds.

For therapeutic and prophylactic strategies against AD, it will be essential to intervene in the pathological progression from Aβ accumulation to tau accumulation at the MCI stage. In fact, a recent study has presented evidence that the positron emission topography (PET) profile of tau predicts cognitive decline in the AD continuum [96]. Thus, suppressing AβO neurotoxicity, which is implicated in the exacerbation of AD pathology at an earlier stage, is a reasonable strategy. In a pathophysiological context, preventing AβO-associated oxidative stress will prove particularly important in suppressing AβO neurotoxicity. The most appealing current therapeutic strategy for AD is immunotherapy using specific Aβ antibodies, and several promising drugs are under development [11,12]. These drugs, however, have disadvantages, such as poor BBB penetration and liability for development of vasogenic edema [97]. AβO-neurotoxicity-reducing agents can be used in combination with these drugs to produce a synergistic therapeutic effect. Importantly, such agents are generally safe without serious side effects, as most are of natural origin. To the best of our knowledge, among the small molecules described above, only RSV has been tested in clinical trials [98]. Hopefully, more clinical trials will be carried out in the near future to test the efficacies of small molecules targeting AβO neurotoxicity. Importantly, it is possible that these antioxidative small molecules might also be beneficial in the treatment of other neurodegenerative disorders, including PD.

## Figures and Tables

**Table 1:** Summary of the small molecules that can reduce AβO neurotoxicity.

Compound	MW	Experimental Models		Route	Ameliorative Effects				Refs
		In Vitro	In Vivo		OS	Apoptosis	Cognitive Impairment	Aβ Accumulation	
Tyrosol	138	Primary neurons	5XFAD	oral	+	+	+	−	[26]
H-Tyr	154		APP/PS1	oral	+	+	+	−	[27,29]
			AβO + ia injection						[28]
Honokiol	266	Primary neurons	AβO injection PS1V97L APP/PS1	ip	+	+	+	+	[38,39,40]
Rhynchophylline	384		AβO injection	ip	+	+	+	n.d.	[42,43]
Astragaloside IV	785	HT22	AβO injection	ip	n.d.	+	+	+	[46]
			APP/PS1						[44]
Ferulic acid	194	LAN5	APP/PS1	oral	+	n.d.	+	+	[49,51,52]
PQM130	381	SH-SY5Y	AβO injection	ip	+	+	+	n.d.	[53,54]
Anthocyanins	450<	HT22	APP/PS1	ip	+	+	+	n.d.	[57]
CAPE	284		AβO injection	ip	+	+	+	n.d.	[61]
Guanosine	283	SH-SY5Y	AβO injection	oral	+	+	+	n.d.	[64,65]
NMN	334	Slice cultures	AβO injection	ip	+	n.d.	+	+	[67]
			APP/PS1	subcutaneous					[68]
Resveratrol	228	SH-SY5Y							[72]
		Primary neurons	APP/PS1	oral	+	n.d.	n.d.	+	[73]

H-Tyr, hydroxytyrosol; CAPE, caffeic acid phenyl ester; NMN, nicotinamide mononucleotide: ia, ibotenic acid; ip, intraperitoneal; MW, molecular weight; OS, oxidative stress; n.d., not determined.
